# Editorial: Education in systems biology 2022

**DOI:** 10.3389/fsysb.2023.1176588

**Published:** 2023-03-13

**Authors:** Edoardo Saccenti

**Affiliations:** Laboratory of Systems and Synthetic Biology, Wageningen University & Research, Wageningen, Netherlands

**Keywords:** Education, systems biology, systems thinking, early-stage researcher, didactic methods

The launch of the Research Topic dedicated to *Education in Systems Biology 2022* (https://www.frontiersin.org/research-topics/28852/education-in-systems-biology-2022#overview) originated from the recognition by the Editorial Board of Frontiers in Systems Biology of the crucial role that education at the graduate and post-graduate level can play in fostering systems thinking through the use of mathematical reasoning and modeling. This Research Topic aimed to emphasize the importance of educating and training students from both biological and mathematical backgrounds in how to think in a systematic way to address modern biological and biomedical problems.

We invited manuscripts that address questions such as effective methods for teaching math and computation applied to biological systems, available software tools for teaching systems biology research, best practices for teaching students with different backgrounds, core concepts of systems science and how these concepts can be taught, and fundamental mathematical tools required for systems reasoning. This Research Topic was also aimed at offering the possibility of publication and research credits for teaching efforts, which are an integral (and often very relevant) part of the activity of academic researchers but are often not acknowledged due to the lack of appropriate journals dedicated to education.

The Research Topic “Education in Systems Biology 2022” published four articles: three were dedicated to teaching strategies and course development in systems biology, while the fourth addressed, from a didactical point, the fundamental problem of parameter estimation.

The article “*Research-driven education: An introductory course to systems and synthetic biology*” by Smith et al., presents an approach to teaching systems and synthetic biology to undergraduate students based on the DBTL (Design-Build-Test-Learn) framework. It introduces a course designed to provide students with hands-on experience in conducting research in these fields as well as to introduce them to the latest tools and technologies used in these areas.

The course presented is structured around a research project in which students work in teams to design and execute experiments, analyze data, and present their findings. The authors highlight the benefits of this approach, including the development of critical thinking skills, the promotion of scientific communication, and the enhancement of scientific literacy.

The article provides an overview of the course structure, including the learning objectives, the research project covered, and the assessment methods used. The authors also discuss the challenges and opportunities associated with implementing this approach, such as the need for interdisciplinary collaboration, the availability of resources, and the importance of providing mentorship and support to students.

The article “*Teaching computational systems biology with an eye on quantitative systems pharmacology at the undergraduate level: Why do it, who would take it, and what should we teach?*” by Androulakis presents the design of a course on computational systems biology as part of a Biomedical Engineering program with a focus on quantitative systems pharmacology at the undergraduate level.

The paper provides an overview of the key concepts and tools used in computational systems biology and quantitative systems pharmacology, such as modeling and simulation, network analysis, and data visualization. It proposes a curriculum for an undergraduate course in computational systems biology and quantitative systems pharmacology that includes topics such as mathematical modeling, systems analysis, and drug discovery. The author also discusses the challenges and opportunities associated with implementing this curriculum, such as the need for recognizing the inherent interdisciplinary of computational systems biology.

The article “*Teaching Systems Biology: Current Practices, Challenges, and Opportunities*” by Voit offers a broad overview of the characteristics of systems biology and advocates for the integration of biological systems thinking into educational programs of disciplines other than biophysics, biochemistry, bioinformatics, and bioengineering, which are inherently related to systems modeling. The author cites medicine, epidemiology, and public health, as well as applied mathematics and computer science, as other areas of research that would benefit from integrating systems thinking into teaching programs. The paper presents examples of how systems thinking can be incorporated into the education curriculum at different levels of complexity and also offers examples of the integration of biological systems thinking into other disciplines.

Finally, the article “*Methods of quantifying interactions among populations using Lotka-Volterra models*” by Davis et al. provides an extensive and complete overview of the use of Lotka-Volterra models to study the interactions among populations in ecological and biological systems. The authors describe the various types of interactions, such as predation, competition, and mutualism, and how these interactions can be mathematically modeled using Lotka-Volterra equations, together with different methods for analyzing the data generated from such models, such as linear regression, model selection, and parameter estimation. The authors highlight the importance of choosing appropriate statistical methods and model parameters to ensure the accuracy and reliability of the results. Several cases are presented that illustrate the application of Lotka-Volterra models in ecological and biological research. These case studies demonstrate the potential of Lotka-Volterra models to provide insights into the dynamics of complex systems and to inform the development of management and conservation strategies, as well as how these models can be used when designing or teaching a course where parameter estimation is presented in the context of systems biology.

The authors also discuss the limitations and challenges associated with using Lotka-Volterra models, such as the assumption of constant parameters and the need for long-term data to capture the dynamics of ecological and biological systems accurately.

To summarize, the articles published in the *Education in Systems Biology 2002* Research Topic discuss various aspects of education in systems and synthetic biology. They highlight the importance of hands-on research projects, interdisciplinary collaboration, and teaching the latest tools and technologies available in these fields, as well as emphasize the benefits of promoting scientific literacy and critical thinking in students. Additionally, they discuss the challenges and opportunities associated with implementing interdisciplinary education and integrating biological systems thinking into education programs of other disciplines.

